# Supplementary material for: Neurotoxicity of Lanthanum Salts: A Narrative Review of Mechanistic Insights from Cellular and Animal Models
Source: Molecules. 2025 Sep 15;30(18):3748. doi: 10.3390/molecules30183748 (PMC12472581; doi:10.3390/molecules30183748)
Supplement: Supplementary file 1 [file molecules-30-03748-s001.zip › molecules-3818584-supplementary.pdf]

molecules

MDPI

Supplementary material

Neurotoxicity of Lanthanum Salts: A Narrative Review of Mechanistic Insights from Cellular and Animal Models

Tudor Mihai Magdas [1,2,3], Constantin Bodolea [4,*], Claudia Gherman [3], Ariana Raluca Hategan [2], Dana Alina Magdas [2], Maria David [2], Roxana Denisa Capras [1] and Gabriela Adriana Filip [1,2]

1 Department of Anatomy and Embriology, "Iuliu Hatieganu" University of Medicine and Pharmacy, 8 Victor Babeş Street, 400012 Cluj-Napoca, Romania; tmmagdas@gmail.com (T.M.M.); capras.roxana@umfcluj.ro (R.D.C.); gabriela.filip@umfcluj.ro (G.A.F.)
2 National Institute for Research and Development of Isotopic and Molecular Technologies, 67-103 Donat Street, 400293 Cluj-Napoca, Romania; ariana.hategan@itim-cj.ro (A.R.H.); alina.magdas@itim-cj.ro (D.A.M.); maria.david@itim-cj.ro (M.D.)
3 Department of Surgery–Practical Abilities, "Iuliu Hațieganu" University of Medicine and Pharmacy, 400337 Cluj-Napoca, Romania; gherman.claudia@umfcluj.ro
4 Anesthesia and Intensive Care Department, "Iuliu Hatieganu" University of Medicine and Pharmacy, 400347 Cluj-Napoca, Romania
* Correspondence: constantin.bodolea@umfcluj.ro

Table S1. Summary table of the experimental studies and key outcomes of the analyzed studies in the present review.

First Author (Year)	Type of Cell/Animal Used	Lanthanum Salt	Dosage Used	Duration of the Experiment	Key Outcomes
Yang (2011) [24]	Primary cultured rat astrocytes	$LaCl_3$	0, 0.25, 0.5, and 1.0 mM $LaCl_3$	12, 24, 48 hours	Induced astrocyte apoptosis via the intrinsic mitochondrial pathway, cytochrome c release, and caspase-9/3 activation.
Wu (2013) [53]	Primary cerebral cortical neurons (Wistar rats)	$LaCl_3$	0, 0.01, 0.1, and 1.0 mM $LaCl_3$	24 hours	Cortical neuron apoptosis driven by increased ROS production, elevated intracellular Ca^{2+}, and Bax/Bcl-2 upregulation.
Zhang (2017) [52]	Primary rat astrocytes	$LaCl_3$	0, 0.125, 0.25, and 0.5 mmol L^{-1} $LaCl_3$	24 hours	Reduced astrocyte viability via oxidative stress resulting from inhibition of the Nrf2 antioxidant response element (ARE) pathway.
Sun (2018) [50]	Primary rat cortical astrocytes and co-cultured neurons	$LaCl_3$	0.25, 0.5, and 1.0 mM $LaCl_3$	24 hours	Impaired astrocyte-neuron lactate shuttle (ANLS) by reducing glucose uptake (↓GLUT1) and lactate transport (↓MCT1/4).
Yan (2019) [54]	BV2 microglial cells and primary mouse cortical neurons	$LaCl_3$	0, 0.05, 0.1, or 0.2 mM $LaCl_3$	24 hours	Activated microglia via the NF-κB pathway, leading to the release of neurotoxic

					factors including NO, TNF-α, and IL-1β.
Wu (2020) [28]	Murine cerebral endothelial cells (bEnd.3) and Wistar rats	$LaCl_3$	0.125, 0.25, and 0.5 mM $LaCl_3$	24 hours	Increased BBB permeability by disrupting endothelial junctions (↓VE-Cadherin) via Ca^{2+}-dependent RhoA/ROCK pathway activation
Song (2021) [48]	Primary cerebral cortical neurons from newborn rats	$LaCl_3$	0.025, 0.05, and 0.1 mM	24 hours	Defective axonal growth by inhibition of the LKB1-STK25-GM130 pathway, affecting Golgi apparatus regulation.
Gao (2019) [49]	Cerebral cortical neurons of neonatal Wistar Rats (Primary cultured neurons)	$LaCl_3$	0, 0.25, 0.5, and 1.0 mM $LaCl_3$	24 hours	Induced excessive autophagy<br>Inhibition of the Akt/mTOR signaling pathway<br>Activation of the AMPK/mTOR signaling pathway
Feng (2006) [30]	Male Wistar rats	$LaCl_3$	0, 0.1, 2, and 40 mg/kg/day of $LaCl_3$ via oral gavage	From 4 weeks through 6 months of age	Hippocampal La accumulation, decreased brain Ca, Fe, Zn; inhibited Ca^{2+}-ATPase & AChE activity; Increased escape latency and total distance traveled in the Morris water maze test.
Feng (2006) [36]	Wistar rats (maternal and offspring)	$LaCl_3$	0, 0.1, 2, and 40 mg/kg/day of $LaCl_3$ via oral administration	From gestation day 0 through 5 months of age	Developmental toxicity - reduced brain DNA concentration, impaired weight gain, and deficits in motor reflexes and endurance.
He (2008) [39]	Wistar rats	$LaCl_3$	0, 0.1, 2, and 40 mg/kg/day of $LaCl_3$ via oral gavage	From maternal gestation until the rats were 6 months of age	Induced oxidative stress, impaired antioxidant defense mechanisms, Ca^{2+} dysregulation; pyramidal cell loss in hippocampal CA3;<br>Increased escape latency and total distance traveled;<br>Impaired acquisition of spatial learning and memory.
Zhao (2010) [51]	Male CD-1 (ICR) mice	$LaCl_3$	20 mg/kg/day of $LaCl_3$ via intraperitoneal injection	14 days	Induced ROS production and decreased antioxidants; increased glutamate and decreased AChE activity
Yang (2013) [47]	Lactational Wistar rats and their offspring	$LaCl_3$	0, 0.25, 0.50, and 1.0% $LaCl_3$ in drinking water	3 weeks (for lactating dams), followed by 3	Stimulated apoptosis in hippocampus via increased $[Ca^{2+}]i$, upregulated

				weeks (via lactation for pups) and an additional 1 month (via drinking water)	Bax/Bcl-2 ratio, and activation of caspases 9 & 3.; Chromatin condensation and nuclear fragmentation in the CA1 area of the hippocampus.
Zheng (2013) [31]	Wistar rats (maternal and offspring)	$LaCl_3$	0, 0.25, 0.50, or 1.00% $LaCl_3$ in drinking water	During pregnancy and lactation, then for 1 month post-weaning	Altered synaptic ultrastructure; downregulated NF-κB signaling and reduced BDNF expression.; Increased escape latency and total distance traveled in memory tests.
Du (2015) [46]	Wistar rats (dams and offspring)	$LaCl_3$	0, 0.25%, 0.5%, and 1.0% $LaCl_3$ in drinking water	From birth, through a 3-week lactation period, then for one month post-weaning	Hippocampal excitotoxicity: elevated glutamate/Ca^{2+}, overactivation of NMDA receptors (NR1/NR2B), and increased NO/cGMP signaling.
Zhang (2016) [43]	Wistar rats (maternal and offspring)	$LaCl_3$	0, 9, 18, or 36 mM $LaCl_3$ (0 to 0.5%) in drinking water	From birth, through 3-week lactation, then for 2 months post-weaning	Oxidative stress (↑MDA, ↓GSH/SOD) via suppression of the Nrf2 pathway and its target genes (HO-1, NQO1); Decreased body and brain weight, poorer spatial long-term memory, and increased escape latency.
Jin (2017) [35]	Wistar rats (maternal and offspring)	$LaCl_3$	0, 0.125, 0.25, 0.5, or 1% $LaCl_3$ in drinking water	During pregnancy and lactation, followed by 1 month of direct exposure to pups after weaning	Disrupted energy metabolism: decreased hippocampal lactate levels and expression of monocarboxylate transporters (MCTs); Impaired spatial memory and learning, but locomotion was not impaired.
Hu (2018) [40]	Wistar rats (maternal and offspring)	$LaCl_3$	0, 0.125, 0.25, and 0.5% $LaCl_3$ in drinking water	From the embryonic phase to one month after weaning	Inhibition of hippocampal long-term potentiation (LTP) linked to impaired glutamate-glutamine cycle and reduced Na^+/K^+-ATPase activity; Increased escape latency and disorganized, purposeless paths in the water maze.
Wang (2020) [41]	Wistar female rats and their offspring	$LaCl_3$	0.25%, 0.5%, and 1.0% $LaCl_3$ in drinking water	During gestation and lactation, then directly to offspring until 1 month after weaning	Increased neuronal apoptosis by inhibiting Akt phosphorylation, leading to increased Bax/Bcl-2 ratio and caspase-3 activation;

					Memory impairment, with increased escape latency and swimming distance.
Sun (2019) [37]	Wistar rats (maternal and offspring)	$LaCl_3$	0%, 0.125%, 0.25%, and 0.5% $LaCl_3$ in drinking water	From conception to the end of lactation (Postnatal Day 20)	Reduced dendritic spine density in CA1 neurons Increased escape latency and total distance traveled; Unaffected swimming speed.
Yu (2020) [42]	Wistar rats (maternal and offspring)	$LaCl_3$	0%, 0.25%, 0.5%, and 1.0% (w/v) $LaCl_3$ in drinking water	From the embryonic phase to 1 month after weaning	Mitochondrial dysfunction: promoted fission (↑Drp1), inhibited fusion (↓Mfn1/2), and induced Parkin-mediated mitophagy; Increased escape latency and total distance traveled; Significantly weaker spatial memory.
Zheng (2020) [47]	Wistar rats and their offspring	$LaCl_3$	0.25%, 0.5%, and 1% $LaCl_3$ in drinking water	To dams before/during lactation, then to offspring for 28 days post-weaning	Induced apoptosis by upregulating miR-124, which directly inhibits the PI3K/Akt survival pathway; Increased escape latency, extended swimming distance, and shorter time spent in the target quadrant.
Yan (2022) [55]	Chinese Kun Ming mice (maternal and offspring)	$LaCl_3$	0, 18, 36, or 72 mM $LaCl_3$ (0 to 1%) in drinking water	During lactation for dams, then for 2 months post-weaning for offspring	Neuroinflammation characterized by microglia activation (↑Iba1) and increased mRNA of pro-inflammatory cytokines (TNF-α, IL-1β, IL-6); Poorer spatial long-term memory and increased escape latency.
Liu (2022) [34]	Wistar rats (maternal and offspring)	$LaCl_3$	0%, 0.125%, 0.25%, and 0.5% $LaCl_3$ in drinking water	During gestation and a 3-week lactation period	Disrupted Mitochondria-Associated Membrane (MAM) integrity, leading to ROS accumulation and AMPK/mTOR-mediated autophagy; Increased escape latency and total distance traveled with chaotic paths; Locomotion was not impaired.
Ding (2021) [45]	Wistar rats (maternal and offspring)	$LaCl_3$	0, 0.25%, 0.5%, and 1.0% $LaCl_3$ in drinking water	From birth, through a 3-week lactation period, then 1 month post-weaning	Induced neuronal apoptosis by inhibiting the PI3K/Akt/mTOR pathway, resulting in decreased HIF-1α and VEGFexpression;

					Damaged avoidance conditioned reflex ability
Song (2022) [48]	Male Wistar rats	$LaCl_3$	2, 20, or 200 mg/kg/day via gavage	90 consecutive days	Impaired axonogenesis by downregulating the LKB1-MARK2 signaling pathway, affecting microtubule stability.
Han (2022) [38]	Caenorhabditis elegans	$La(NO_3)_3$	Concentrations based on the 48-h LC_{50} (93.163 mg/L for L1, 648.0 mg/L for L4)	48 hours	Dopaminergic & GABAergic neurodegeneration ROS production Aggregation of α-synuclein; Inhibited movement behavior; Inhibited growth and development.
Xiao (2021) [27]	Male Wistar rats	$La(NO_3)_3$	0, 2, 20, or 200 mg/kg/day via gavage	30 consecutive days, post-weaning	Decreased plasma acetylcholine and norepinephrine; Neuron loss in hippocampal CA1; Impaired spatial learning and memory; Reduce strength.
